# Phytochemical Profile and Nutraceutical Value of Old and Modern Common Wheat Cultivars

**DOI:** 10.1371/journal.pone.0045997

**Published:** 2012-09-26

**Authors:** Emanuela Leoncini, Cecilia Prata, Marco Malaguti, Ilaria Marotti, Antonio Segura-Carretero, Pietro Catizone, Giovanni Dinelli, Silvana Hrelia

**Affiliations:** 1 Department of Biochemistry, G. Moruzzi Alma Mater Studiorum, University of Bologna, Bologna, Italy; 2 Department of Agroenvironmental Science and Technology, Alma Mater Studiorum, University of Bologna, Bologna, Italy; 3 Department of Analytical Chemistry, University of Granada, Granada, Spain; Vanderbilt University, United States of America

## Abstract

Among health-promoting phytochemicals in whole grains, phenolic compounds have gained attention as they have strong antioxidant properties and can protect against many degenerative diseases. Aim of this study was to profile grain phenolic extracts of one modern and five old common wheat (*Triticum aestivum* L.) varieties and to evaluate their potential antiproliferative or cytoprotective effect in different cell culture systems.

Wheat extracts were characterized in terms of antioxidant activity and phenolic composition (HPLC/ESI-TOF-MS profile, polyphenol and flavonoid contents). Results showed that antioxidant activity (FRAP and DPPH) is mostly influenced by flavonoid (both bound and free) content and by the ratio flavonoids/polyphenols. Using a leukemic cell line, HL60, and primary cultures of neonatal rat cardiomyocytes, the potential antiproliferative or cytoprotective effects of different wheat genotypes were evaluated in terms of intracellular reactive oxygen species levels and cell viability. All tested wheat phenolic extracts exerted dose-dependent cytoprotective and antiproliferative effects on cardiomyocytes and HL60 cells, respectively. Due to the peculiar phenolic pattern of each wheat variety, a significant genotype effect was highlighted. On the whole, the most relevant scavenging effect was found for the old variety Verna. No significant differences in terms of anti-proliferative activities among wheat genotypes was observed.

Results reported in this study evidenced a correspondence between the *in vitro* antioxidant activity and potential healthy properties of different extracts. This suggests that an increased intake of wheat grain derived products could represent an effective strategy to achieve both chemoprevention and protection against oxidative stress related diseases.

## Introduction

Numerous studies confirm that cereals exert a protective action on human health and are key components of a healthy and balanced diet. According to nutritional guidelines, cereals and cereal products are placed at the base of the food pyramid [1]. Epidemiological studies have correlated the intake of whole grains and whole-grain products with a reduced incidence of chronic disease such as cardiovascular disease, diabetes and cancer [2], [3], [4], [5], [6], [7]. The involvement of reactive oxygen species (ROS) in the aetiology of these degenerative conditions has suggested that phytochemicals with antioxidant activity may contribute to patho-prevention [8]. The wide array of phytochemicals present in cereal grains, has been indicated to be responsible for these health-promoting effects [9].

Recent studies on the health benefits of functional products from wheat have become increasingly more focused on the importance of introducing phytochemicals through the use of different varieties. As a consequence, there is a renewed interest in the ancient varieties, particularly with regard to potential nutraceutical qualities [10], [11], [12], [13]. Conceptually an old wheat cultivar can be defined as a not dwarf and unregistered genotype, while a modern one can be considered a dwarf or semi-dwarf registered genotype. It is noteworthy that modern breeding programs for genetic improvement have been primarily focused on yield improvement, and on the improvement of disease and pest resistance rather than nutritional and functional characteristics. As a result, little attention has been given to the selection of varieties according to nutritional value. Therefore, research aiming at the characterization of the phytochemical profile of wheat varieties focusing on antioxidants compounds like polyphenols, flavonoids, carotenoids, tocopherols, may represent a new prospect for the genetic improvement of the genus *Triticum*.

The characterization of bioactive components present in staple food represents an essential step in the validation process of such nutritional strategies and it is central for specific “functional” food definition [14]. The identification of bioactive components present in food consumed on a daily basis, represents an essential step in the design of a functional food aimed at the prevention of chronic-degenerative diseases. The word nutraceutical (a combination of nutrition and pharmaceutical) indicates a food component that provides health benefits, including the prevention of diseases. Different experimental approaches are commonly used for nutraceutical value assessment of foodstuff and ingredients. The use of *in vitro* cell model systems represents a powerful and informative tool for the definition of the antioxidant capacity and related cytoprotective effects [15].

The objectives of this study were to characterize the polyphenol and flavonoid profiles of different wheat varieties and to correlate their antioxidant activity, due to a peculiar phytochemicals composition, to nutraceutical effects exerted in biological systems. In particular, the potential effect of different extracts (derived from old and modern wheat varieties) in counteracting ROS production or decreasing ROS basal levels was studied in two model systems. The evaluation of the potential cardioprotective effect was conducted using primary cultures of neonatal rat cardiomyocytes exposed to oxidative stress. The antiproliferative effect in transformed cells was studied by means of HL60 cell line. The analysis was focused on the influence exerted by the selected extracts on cell viability and intracellular ROS production and levels. Data obtained in this study revealed a significant genotype influence on antioxidant activity of wheat extracts and this effect could be ascribed to the peculiar phenolic pattern of each wheat variety. Although further intervention studies in humans are required, *in vitro* studies represent an essential step for health claim substantiation regarding functional properties of the wheat varieties analyzed in this study.

## Materials and Methods

### Chemicals and reagents

All chemicals utilized were of the highest analytical grade and were purchased from Sigma Aldrich (St Louis, MO, USA) unless otherwise stated.

### Grain samples

Wheat samples included 5 old, not dwarf and unregistered Italian genotypes (Andriolo, Frassineto, Gentil rosso, Inallettabile, Verna) and one modern, dwarf registered cultivar (Palesio) of common wheat (*Triticum aestivum* L.). Seeds from all of the investigated genotypes were grown in the same location at the experimental farm of the University of Bologna, Cadriano (latitude 44°33′N, longitude 11°21′E, 32 m a.s.l.), Italy, during the growing season 2006/2007. Each genotype was grown in plots (6×5 m) according to a low input agro-technique (nitrogen fertilization with 10 kg NO_3_ ha^−1^ applied in pre-sowing and 20 kg NO_3_ ha^−1^ applied in leaf sheaths lengthening stage). Weeds were hand controlled and no herbicide (or other pesticide) treatment was applied. Plants were harvested at grain full ripening stage. Whole grain samples were milled into flours, immediately cooled to −20°C and kept at this temperature until analysis.

### Preparation of wheat phenolic extracts

Free phenolic compounds were extracted as previously described [16], [17]. Briefly, 1 g of whole wheat flour was mixed with 20 mL of 80% chilled ethanol for 10 min. After centrifugation at 2500× *g* for 10 min, the supernatant was removed and extraction was repeated. Supernatants were pooled, evaporated at 45°C to less than 5 mL, and reconstituted in 10 mL of water. The residue from the free phenolic extraction was subjected to alkaline and acid hydrolysis to recover the bound phenolic compounds [18]. For each wheat variety, the free and bound phenolic fractions were gathered and stored at −40°C until use.

In order to exclude the presence of other antioxidant compounds in the phenolic extracts prepared as described above, the content of carotenoids, tocopherols, tocotrienols and ascorbic acid was evaluated. Carotenoid content, measured according to [19], ranged between 0.2–0.01 µg/mL. HPLC/MS analysis, performed as in [12], confirmed the absence of both tocopherols/tocotrienols and ascorbic acid and the presence of negligible amount of carotenoid derivatives (sintaxanthin, bixin, octadecyl ester, 8′-Apo-β-carotenal, 15,15′-didehydro) and of fatty acids, in all wheat varieties.

### Total polyphenol and flavonoid contents

The amount of total phenolics in extracts (free and bound) was determined according to the Folin–Ciocalteu procedure [20]. Results are expressed as micromoles of gallic acid equivalents (GAE) per 100 g of grain. Total flavonoid content was determined according to a colorimetric method described previously by Adom *et al*. [16]. Briefly, appropriate dilutions of sample extracts were reacted with sodium nitrite, followed by reaction with aluminium chloride to form a flavonoid–aluminium complex. Solution absorbance at 510 nm was immediately measured and compared to that of catechin standards. Flavonoid content was expressed as micromoles of catechin equivalent (CE) per 100 g of grain.

### Antioxidant activity

The total antioxidant activity of wheat phenolic extracts was measured by ferric ion reducing power (FRAP) assay [21] and 2,2-diphenyl-1-picrylhydrazyl radical (DPPH•) assay [22] with some modifications. Briefly, freshly prepared FRAP Reagent (10∶1∶1 Acetate buffer 300 mM pH 3.6, 10 mM 2, 4, 6-tripyridyl-s-triazine (TPTZ) in 40 mM HCl and 20 mM FeCl_3_*6H_2_O) was mixed with the sample and allowed to stand for 60 min at room temperature before measuring absorption at 593 nm. Aqueous solutions of Fe^2+^ (FeSO_4_*6H_2_O) in the concentration range of 125–1250 µmol/L were used for calibration of the FRAP assay. FRAP values were expressed as millimoles of Fe^2+^ per 100 g of sample (mmol of Fe^2+^/100 g of grains FW). As regards DPPH assay, results were expressed as micromoles of Trolox equivalent/g of grain FW.

### Characterization of phytochemical profile of phenolic compounds in modern and old varieties

HPLC analysis was performed using an Agilent 1200-RRLC system (Agilent Technologies, CA, USA) consisting of a vacuum degasser, autosampler, a binary pump and a UV–Vis detector. Phenolic compounds were separated using a RP C18 analytical column (4.6 mm×150 mm, 1.8 µm particle size) from Agilent ZOR-BAX Eclipse plus. The mobile phases and gradient program used were as previously described [12]. The effluent from the HPLC column was splitted using a T-type phase separator before being introduced into the mass spectrometer (split ratio = 1∶3). The HPLC system was coupled to a microTOF (Bruker Daltonics, Bremen, Germany), an orthogonal-accelerated TOF mass spectrometer (oaTOFMS), equipped with an ESI interface. Parameters for analysis were set using negative ion mode with spectra acquired over a mass range from m/z 50 to 1000. The accurate mass data of the molecular ions were processed through the software Data Analysis 4.0 (Bruker Daltonics, Bremen, Germany), which provided a list of possible elemental formula by using the Smart Formula Editor. Confirmation of elemental compositions was based on isotopic abundance patterns and on external instrument calibration as reported in Dinelli *et al*. [12].

### Cell model systems

In order to assess whether there is correspondence between the *in vitro* antioxidant activity and potential healthy properties of different extracts, two cell models were used: a leukemia cell line, HL60, and primary cultures of neonatal rat cardiomyocytes. Cells received different concentrations of wheat extract for 24 hours after which their effects were evaluated in terms of intracellular ROS production and cell viability.

### Cell cultures and treatments

Cultured rat cardiomyocytes were prepared as reported by Angeloni *et al*. [23]. The investigation conforms with the Guide for the Care and Use of Laboratory Animals published by the US National Institutes of Health (NIH Publication No. 85–23, revised 1996) and have been approved by the Ministry of Health, Rome, Italy and by the University of Bologna Ethics Committee. Cells, seeded at a concentration of 5×10^5^/mL in 96 well plates, were grown until complete confluence. Acute myeloid leukaemia HL60 cells were grown as reported by Prata *et al*. [24]. Cells were treated with different concentrations of wheat extracts (as reported in figure legends) for 24 h, and with equivalent concentrations of 70% ethanol alone.

### Measurement of intracellular reactive oxygen species (ROS) levels

The formation of ROS was evaluated using a fluorescent probe, 2′,7′-dichlorofluorescin diacetate (DCFH-DA), as described by Angeloni *et al*. [25]. Briefly, after 24 h treatment with wheat extracts cells were incubated with 5 µM DCFH-DA for 30 min at 37°C and analyzed spectrofluorimetrically at λ_Ex/Em_ 485/535 nm in a multiwell plate reader VICTOR3 V™ Multilabel Counter (Perkin Elmer). In cultured cardiomyocytes oxidative stress was induced by 100 µM H_2_O_2_ in PBS for 30 min. Data were expressed as the percentage of inhibition of intracellular ROS produced by H_2_O_2_ exposure. In HL60 cells, fluorescence values represent the percentage of inhibition of intracellular ROS in respect to controls.

### Measurement of cell viability

Cell viability in the absence/presence of wheat extracts was measured using the MTT assay. Cells were incubated with MTT solution (0.5 mg/mL). MTT reduction product was solubilized in lysing buffer and analysed spectrophotometrically at λ 570 nm in a microplate reader VICTOR3 V™ Multilabel Counter (Perkin Elmer). In cultured cardiomyocytes cell viability was evaluated after induction of oxidative stress by 100 µM H_2_O_2_ in PBS for 30 min. Data were expressed as percentage of viable cells with respect to controls.

### Statistical analysis

Statistical analyses were performed using STATISTICA Software v. 7.1, (StatSoft, Tulsa, Oklahoma, USA). Levels of statistical significance were evaluated using one-way analysis of variance (ANOVA) and two-way ANOVA followed by Fisher's LSD post test for chemical analyses and *in vitro* tests, respectively. Chemical analyses were performed on three replicates for each wheat variety; four independent experiments, each in triplicate format, were performed in cell model systems. Values are represented as means ± SEM. Values of *p*<0.05 were considered to be statistically significant. Cluster analysis (Unweighted Pair Group Average Method with arithmetic averaging – UPGMA) was carried out on phenolics composition data obtained by HPLC - ESI – TOF and on Manhattan distance matrix to seek for hierarchical association among the wheat varieties. PCA is an unsupervised clustering method, which is a powerful tool for analysis of multivariate data, without requiring any knowledge of the dataset [26]. PCA was used to transforms a number of correlated variables into a smaller number of uncorrelated variables called principal components [27]. In the present study, the correlation method was preferred over covariance since PCA on the covariance matrix is not invariant to a component-wise change of scale [28]. With this method the original space for variable measurements was projected down onto two low-dimensional subspaces. One of these was case-related (six common wheat genotypes), the other was variable-related. The eleven variables were FRAP activities of free and bound polyphenol fractions, DPPH activities of free and bound polyphenol fractions, ratio of total flavonoids on total polyphenols, total number of free and bound polyphenol species (including isomeric forms), MTT viability test on cardiomyocytes and leukemia cell line, ROS test on cardiomyocytes and leukemia cell line. The variable-related subspace was analyzed (factor loading) to understand the correlation between the variables and factors (principal component).

## Results

### Polyphenol and flavonoid content and antioxidant activity of wheat varieties

The polyphenol content (free, bound and total fractions) of each wheat variety is presented in Table 1 and expressed as mg of gallic acid equivalent (GAE) per 100 g of grain. Free polyphenol content ranged from 29.04 mg GAE/100 g of grain (Palesio) to 50.16 mg GAE/100 g of grain (Verna). Along with Verna, Andriolo and Frassineto showed high free polyphenol contents. As regards the bound fraction, phenolic content ranged from 104.21 mg GAE/100 g of grain (Palesio) to 129.03 mg GAE/100 g of grain (Frassineto). Most comparisons of bound phenolic content among investigated varieties were not statistically different (*p*>0.05), except Frassineto that displayed higher phenolic content than Palesio (*p*<0.05). Frassineto had high total phenolic content (173.48 mg GAE/100 g of grain) which was similar to Andriolo, Gentil rosso, Inallettabile and Verna (*p*>0.05) but higher than Palesio (133.25 mg GAE/100 g of grain).

**Table 1:** Polyphenol and flavonoid content in wheat grains.

Cultivar	FPC	BPC	TPC	FFC	BFC	TFC
	(mgGAE/100 g)	(mgGAE/100 g)	(mgGAE/100 g)	(mgCE/100 g)	(mgCE/100 g)	(mgCE/100 g)
AN (O)	44.29±2.25 (ab)	126.49±6.05 (ab)	170.78±8.29 (a)	20.05±1.22 (c)	12.53±0.64 (c)	32.58±1.86 (d)
FR (O)	44.45±1.73 (ab)	129.03±8.59 (a)	173.48±6.86 (a)	27.87±1.40 (b)	21.46±1.15 (b)	49.33±0.25 (b)
GR (O)	39.22±2.23 (b)	122.67±4.14 (ab)	161.89±6.36 (a)	11.43±0.25 (d)	19.03±0.51 (b)	30.46±0.76 (d)
IN (O)	40.49±2,23 (b)	111.53±3.82 (ab)	152.02±1.59 (ab)	23.11±1.07 (c)	16.23±0.02 (bc)	39.34±1.07 (c)
VE (O)	50.16±2.70 (a)	116.30±9.23 (ab)	166.46±11.93 (a)	31.96±1.15 (a)	35.73±3.95 (a)	67.69±2.80 (a)
PA (M)	29.04±1.59 (c)	104.21±2.23 (b)	133.25±3.82 (b)	9.90±0.51 (d)	10.24±0.13 (c)	20.14±0.64 (e)

The polyphenol content is expressed as mg gallic acid equivalent/100 g of whole flour and the flavonoid content is expressed as mg catechin equivalent/100 g whole flour.

AN, Andriolo; FR, Frassineto, GR, Gentil rosso; IN, Inallettabile; VE, Verna; PA, Palesio. FPC, free phenolic compounds; BPC, bound phenolic compounds; TPC, total phenolic compounds; FFC, free flavonoid compounds; BFC, bound flavonoid compounds; TFC, total flavonoid compounds; M, modern cultivar; O, old cultivar.

Values are expressed as means ± SEM for triplicates. Means in the same column with common letters are not significantly different (*p*<0.05).

Flavonoid content of wheat samples is presented in Table 1 and expressed as mg of catechin equivalent (CE) per 100 g of grain. Significantly different values were observed among the 6 tested wheat varieties in the free, bound and total flavonoid fractions. Free flavonoid content ranged from 9.90 to 31.96 mg CE/100 g of grain for Palesio and Verna, respectively. Verna had the highest free flavonoid content and this level was significantly different from that of Frassineto, Andriolo and Inallettabile. Gentil rosso and Palesio had the lowest free phenolic contents. The bound flavonoid content ranged from 10.24 (Palesio) to 35.73 (Verna) mg CE/100 g of grain. Verna had the highest bound flavonoid content, followed by Frassineto, Gentil rosso and Inallettabile with similar bound flavonoid values. Palesio and Andriolo had the lowest bound flavonoid content among all tested varieties. The total flavonoid content significantly differed depending on wheat cultivar and it decreased in the order:

Verna > Frassineto > Inallettabile > Andriolo > Gentil rosso > Palesio.

DPPH and FRAP assays were used to measure the antioxidant activity of free, bound and total wheat phenolic fractions (Table 2). Scavenging of DPPH radical allows evaluation of the hydrogen-donating potency of phenolic compounds [29], whereas the FRAP assay measures the reduction of Fe^3+^ (ferric iron) to Fe^2+^ (ferrous iron) giving the concentration of electron-donating antioxidants [21]. The DPPH radical scavenging activities were expressed as micromoles of Trolox equivalents (TE) per gram of wheat grain. All wheat cultivars showed similar antioxidant capacity when considering bound and total phenolic extracts. As regards the free phenolic fractions, Verna extracts exhibited stronger activity to react with and quench DPPH radicals (2.11 µmol TE/g grain) than Palesio extracts (1.16 µmol TE/g grain). In contrast, the antioxidant capacity determined by the FRAP method varied significantly among the varieties in all three phenolic fractions. In the total phenolic fractions FRAP values ranged from 1.11 to 1.45 mmol Fe ^2+^/100 g and decreased in the order: Verna > Andriolo, Frassineto > Gentil rosso > Inallettabile > Palesio. In the free and bound fractions Verna confirmed the highest FRAP values, whereas Inallettabile and Palesio had the lowest antioxidant activity.

**Table 2:** Antioxidant capacity of free and bound fractions of wheat phenolic extracts determined by the DPPH (µmol Trolox equivalent/g grain) and FRAP (mmol Fe^2+^/100 g grain) methods.

	DPPH			FRAP		
	(µmol Trolox equivalent/g grain)			(mmol Fe ^2+^/100 g grain)		
	Free	Bound	Total	Free	Bound	Total
AN (O)	1.90±0.06 (ab)	7.20±1.11 (a)	9.10±1.16 (a)	0.32±0.04 (a)	0.99±0.01 (c)	1.31±0.03 (b)
FR (O)	1.39±0.10 (bc)	7.04±0.96 (a)	8.43±1.06 (a)	0.23±0.02 (b)	1.08±0.01 (a)	1.31±0.03 (b)
GR (O)	1.94±0.20 (ab)	8.05±1.05 (a)	9.99±1.25 (a)	0.20±0.01 (bc)	1.06±0.03 (ab)	1.26±0.04 (b)
IN (O)	1.58±0.11 (abc)	6.35±1.15 (a)	7.93±1.27 (a)	0.12±0.01 (d)	1.01±0.01 (bc)	1.13±0.01 (c)
VE (O)	2.11±0.35 (a)	7.66±0.36 (a)	9.77±0.72 (a)	0.34±0.01 (a)	1.11±0.03 (a)	1.45±0.03 (a)
PA (M)	1.16±0.04 (c)	5.61±0.88 (a)	6.77±0.92 (a)	0.16±0.01 (cd)	0.95±0.01 (c)	1.11±0.01 (c)

AN, Andriolo; FR, Frassineto, GR, Gentil rosso; IN, Inallettabile; VE, Verna; PA, Palesio; M, modern cultivar; O, old cultivar. Values are expressed as means ± SEM for triplicates. Means in the same column with common letters are not significantly different (*p*<0.05).

**Figure 1:**
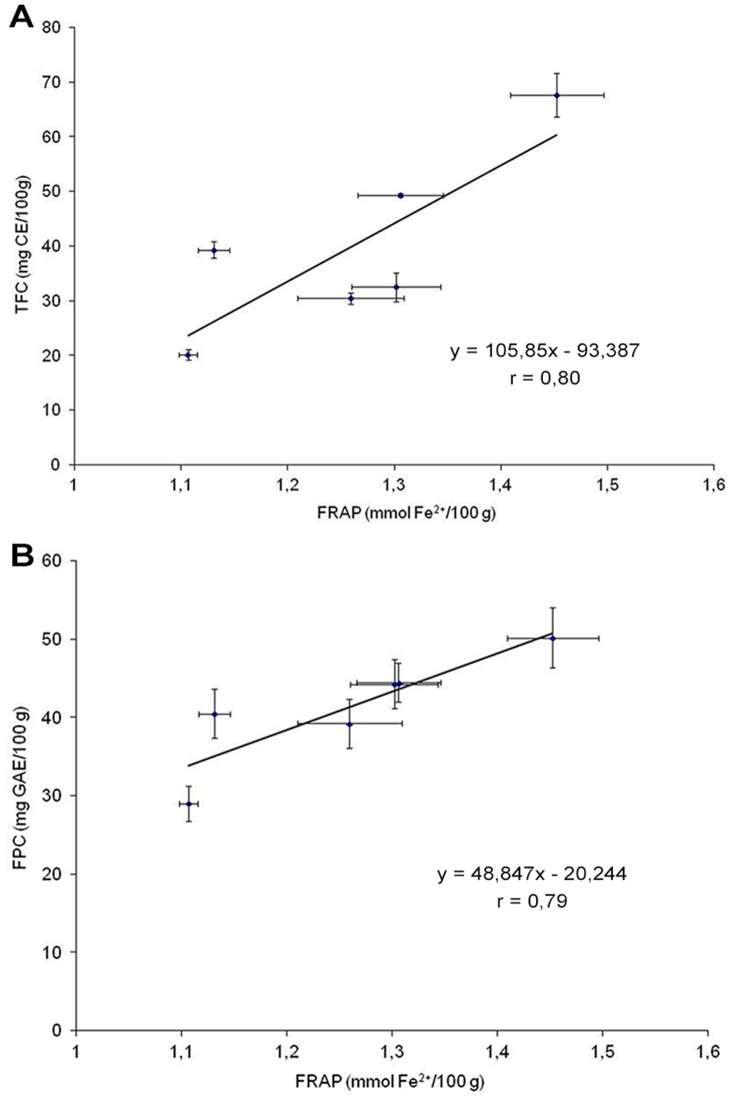
Simple linear correlation of FRAP antioxidant activity with total flavonoid and free polyphenol content in wheat extracts. The antioxidant activity measured by FRAP assay was linearly correlated to total flavonoid (panel A) and free polyphenol contents (panel B) of different wheat varieties analyzed in this study. Data reported in the graphs represent means +/− SD of FRAP activity (horizontal bars) and of total flavonoids/free polyphenols (vertical bars) in each extract. R values are significant at *p*<0.05.

The Antioxidant activity measured by FRAP assay significantly correlated with total flavonoid content (0.80; *p*<0.05) and with free polyphenol content (0.79; *p*<0.05), as presented in Figure 1, whereas no correlation was found between DPPH values and antioxidant phytochemical content of wheat varieties. On average, bound phytochemicals contributed >80% of total antioxidant activity (considering both FRAP and DPPH values) with the exception of Andriolo and Verna, for which the contribution was slightly lower (78% on average).

### Analysis of phenolic compounds in wheat grains

Tentative identifications of phenolic compounds in both free and bound wheat extracts were generated based on elemental composition data determined from accurate mass measurements and comparison with literature data. A total of 17 phenolic compounds were identified and grouped into chemical classes. Compound identification and their occurrence in investigated wheat varieties is presented in Table 3.

**Table 3:** Phenolic compounds detected by HPLC–ESI–TOF-MS in the free and bound extracts of wheat varieties.

No.	Molecular formula	[M-H]^−^	Compound	Class	Sample		Reference
					Free extract	Bound extract	
1	C_9_H_10_O_5_	197.0455	Syringic acid	Phenolic acid	AN FR GR PA	AN FR IN PA	[54]
2	C_8_H_8_O_4_	167.0349	Vanillic acid	Phenolic acid	AN FR GR VE	AN FR IN GR	[54]
3	C_26_H_28_O_15_	579.1355	Lucenin-1/3(luteolin-6/8-C-xyloside-8/6-C-glucoside)	Flavone-C-glycoside	AN FR IN GR VE PA		[55]
4	C_27_H_30_O_15_	593.1511	Vicenin-2 (apigenin-6,8-di-C-glucoside)	Flavone-C-glycoside	AN FR IN GR VE PA		[55]
5	C_26_H_28_O_14_	563.1395	Apigenin-6-C-arabinoside-8-C-hexoside (schaftoside/isoschaftoside)	Flavone-C-glycoside	AN FR IN GR VE PA	AN FR IN GR VE PA	[56]
6	C_25_H_26_O_13_	533.1300	Glycosylated and acetylated 3′,4′,5′-trihydroxy-3,7-dimethylflavone	Flavone-O-glycoside	AN FR IN GR VE PA		[57]
7	C_9_H_8_O_3_	163.0400	p-Coumaric acid	Phenolic acid		AN FR GR VE PA	[54]
8	C_26_H_32_O_12_	535.1821	Pinosylvin(double glycosylation)	Stilbenes	GR VE	AN FR GR VE	[57]
9	C_27_H_30_O_14_	577.1562	Isovitexin-2″-O-rhamnoside	Flavone-C-glycoside	AN FR IN GR VE PA		[55]
10	C_21_H_22_O_8_	401.1241	Glycosylated pinosylvin	Stilbenes		AN FR IN GR VE PA	[57]
11	C_10_H_10_O_4_	193.0506	Ferulic acid	Phenolic acid	FR IN GR VE	AN FR IN VE PA	[54]
12	C_28_H_32_O_15_	607.1668	Methylisoorientin-2″-O-rhamnoside	Flavone-C-glycoside	AN FR GR VE PA		[55]
13	C_23_H_24_O_12_	491.1195	Glycosylated 3′,4′,5′-trihydroxy-3,7-dimethylflavone	Flavone-O-glycoside	AN FR IN GR VE PA		[57]
14	C_20_H_18_O_8_	385.0928	Dihydroferulic acid	Phenolic acid		AN FR IN GR VE PA	[18]
15	C_30_H_26_O_12_	577.1351	Procyanidin B-3	Proanthocyanidin		AN FR GR VE PA	[58]
16	C_20_H_18_O_6_	353.1030	Hinokinin	Lignan		AN IN GR PA	[11]
17	C_20_H_22_O_6_	357.1343	Pinoresinol	Lignan		FR GR	[11]

AN, Andriolo; FR, Frassineto, GR, Gentil rosso; IN, Inallettabile; VE, Verna; PA, Palesio.

Five phenolic acids were observed in most of the wheat varieties: syringic acid (compound 1), vanillic acid (compound 2) and ferulic acid (compound 11) were detected in both free and bound extracts, whereas p-coumaric acid (compound 7) and dihydroferulic acid (compound 14) were exclusively observed in the bound phenolic fractions. Of notes, in free and bound fractions vanillic, syringic and p-cumaric acids were not found in Palesio, Verna and Inallettabile, respectively.

Flavones resulted the most representative flavonoid group of the investigated wheat grains, comprising of C-glycosylated and O-glycosylated compounds. The interpretation of the mass spectra allowed the detection of five C-glycosylated flavones: lucenin-1/3 (luteolin-6/8- C-xyloside-8/6-C-glucoside) (compound 3), vicenin-2 (apigenin-6,8-di-C-glucoside) (compound 4) isovitexin-2″-O-rhamnoside (compound 9) and methyl isoorientin-2″-O-rhamnoside (compound 12) were detected exclusively in the free phenolic fractions of all the wheat varieties, except for methyl isoorientin-2″-O-rhamnoside that was not present in Inallettabile. Apigenin-6-C-arabinoside-8-C-hexoside (schaftoside/isoschaftoside) (compound 5) was found in both free and bound fractions of all the wheat genotypes. Two flavone-O-glycosides were found in the free extracts of all the wheat samples, with masses 491.12 and 533.13, putatively identified as glycosylated 3′,4′,5′-trihydroxy-3,7-dimethylflavone (compound 13) and glycosylated and acetylated 3′,4′,5′-trihydroxy-3,7-dimethylflavone (compound 6), respectively. Two stilbenes were also identified: the glycosylated form of pinosylvin (mass 402.124, C_21_H_22_O_8_) (compound 10) was detected in the bound extracts of all six investigated wheat varieties; compound 8 with mass 535.182 and deduced molecular formula C_26_H_32_O_12_ was tentatively identified as double glycosylated pinosylvin and detected in the free and bound extracts of Verna and Gentil rosso, as well as in the bound fractions of Andriolo and Frassineto.

**Figure 2:**
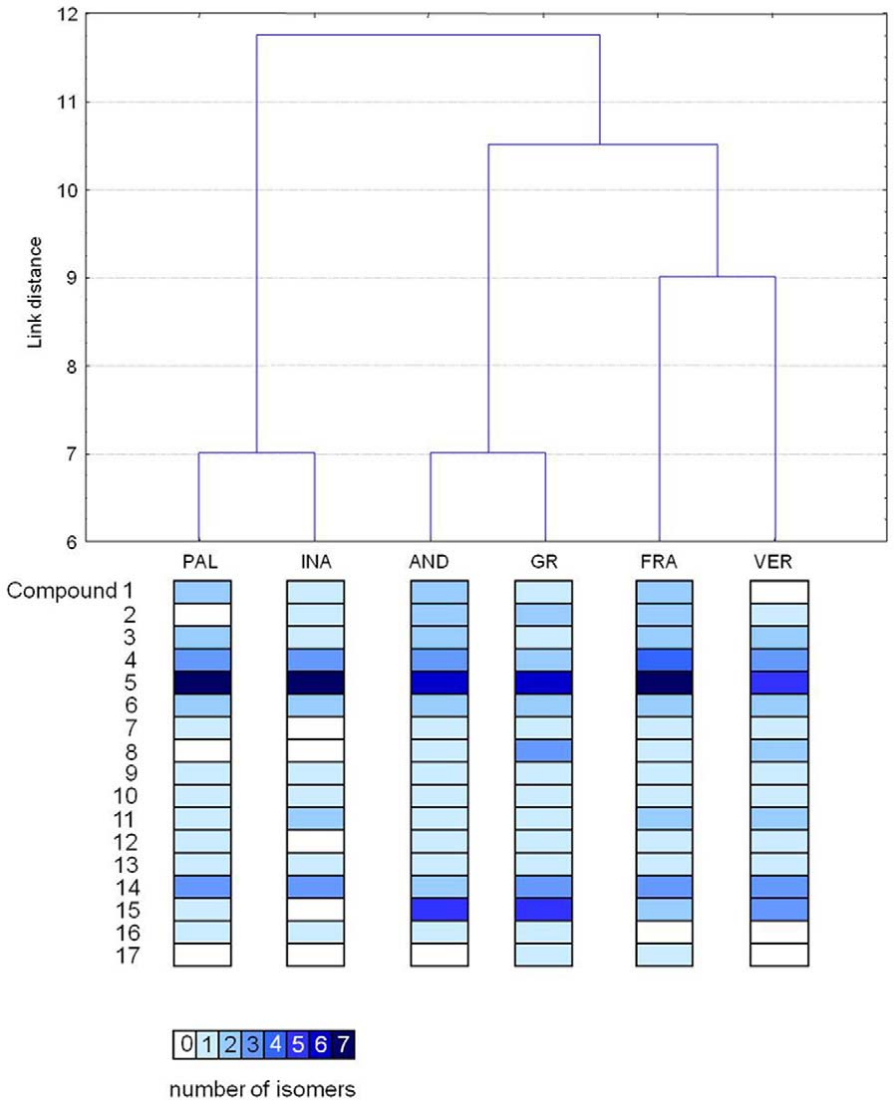
Dendrogram of hierarchical cluster analysis of wheat phenolic composition based on Manhattan similarity distance. For each wheat cultivar, compounds as listed in Table 1 were ordered in columns from the top (compound 1) to the bottom (compound 17). Color intensity, from white to dark blue, indicates the relative abundance of each compound.

One proanthocyanidin (compound 15) with mass 577.135 and deduced molecular formula C_30_H_26_O_12_ was assigned as procyanidin B-3 and detected in the bound extracts of all the wheat genotypes except Inallettabile. Lignan compounds were identified exclusively in the bound phenolic fractions of investigated wheat varieties. Compound 16 with mass 353.103 and deduced molecular formula C_20_H_18_O_6_ was tentatively identified as hinokinin and detected in Andriolo, Inallettabile, Gentil rosso and Palesio varieties. Another lignan compound was identified in Frassineto and Gentil rosso and assigned as pinoresinol (compound 17) with mass 357.135 (C_20_H_22_O_6_). Table 4 shows the total number of different phenolic compounds, including isomer forms, found in both free and bound fractions. No significant differences were observed in the mean number of free, bound and total polyphenol isoforms among all wheat varieties with the exception of Inallettabile which showed the lowest number of compounds. In Figure 2 the different wheat genotypes are clustered according to their phenolic composition determined by HPLC-MS. The dendrogram showed two main divergent groups, the first one formed by Palesio and Inallettabile and the second one including all other genotypes. The latter cluster evidenced two well defined subgroups which included Andriolo and Gentil rosso in the first subgroup and Verna and Frassineto in the second one.

**Table 4:** Total compounds, including isomers, detected in the free and bound phenolic fractions of wheat grains.

Cultivar	NPF	NPB	NPT
Andriolo (O)	16.0±1.0 (ab)	16.0±1.0 (a)	32.0±2.0 (a)
Frassineto (O)	18.0±1.0 (a)	15.0±1.0 (ab)	33.0±2.0 (a)
Gentil rosso (O)	17.0±2.0 (ab)	16.0±1.0 (a)	33.0±3.0 (a)
Inallettabile (O)	13.0±1.0 (b)	11.0±1.0 (c)	24.0±2.0 (b)
Verna (O)	16.0±1.0 (ab)	12.0±1.0 (bc)	28.0±0.0 (ab)
Palesio (M)	15.0±1.0 (ab)	12.0±0.0 (bc)	27.0±1.0 (ab)

NPF, number of free phenolic compounds; NPB, number of bound phenolic compounds; NPT, total number of phenolic compounds; M, modern cultivar; O, old cultivar.

Values are expressed as means ± SEM for triplicates. Means in the same column with common letters are not significantly different (*p*<0.05).

### Antioxidant activity and antiproliferative effects in HL60 cell line

**Figure 3:**
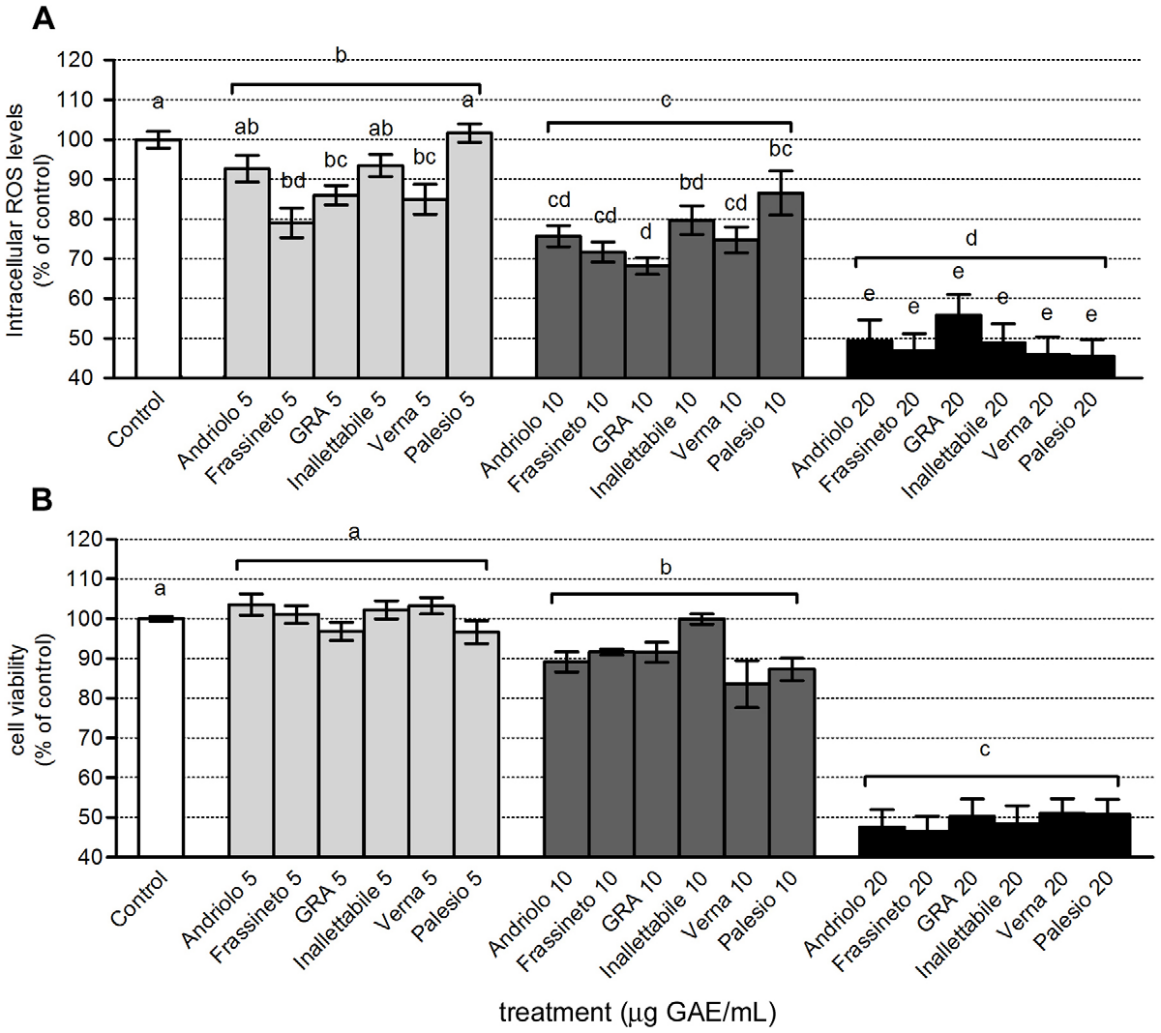
Evaluation of intracellular ROS production and cell viability in HL60 cell line. HL60 cells were treated with different concentrations of phenolic extracts of different wheat varieties (5, 10, 20 µg GAE/mL) for 24 h and intracellular ROS production (A) and cell viability (B) were analyzed as reported in Material and Methods. Data, expressed as % of control, represent mean +/− SEM of 4 independent experiments, each in triplicate. Statistical analysis of differences was carried out by two way ANOVA followed by Fisher's LSD as post test. Different letters represent statistical significance: *p*<0.05.

HL60 cell line, as most cancer cells, is characterized by a high production of ROS, which represents for these cells a proliferative stimulus [30]. Data reported in Figure 3A represent wheat extract ability to reduce intracellular ROS production in HL60 cells. Statistical analysis revealed that the effect of the dose was highly significant (*p*<0.001). The dose 5 µg GAE/mL was significantly different from control with an average reduction in ROS production of 10.3% compared to control. Increasing the dose, there was a linear increase of cellular effects of wheat extracts (R^2^ = 0.999), in fact, doubling the dose (10 µg GAE/mL) resulted in a decreased ROS production of 23.9% compared to control, while 20 µg GAE/mL corresponded to 51.2% decrease in intracellular ROS levels. The genotype did not affect wheat extract antioxidant capacity, meaning that all genotypes exhibited, on average, the same overall reduction of intracellular ROS production. However, since the interaction genotype x dose was statistically significant (*p*<0.05), it is evident that at the different doses tested in our model system not all genotypes maintain the same proportional dose-dependent effect. This is particularly evident at 5 and 10 µg GAE/mL. The modern variety Palesio was not significantly different from control untreated cells, at the lowest dose tested while old varieties Andriolo and Inalettabile partially differed. Only old varieties Frassineto, Verna and Gentil rosso were significantly different compared to controls. At the dose 10 µg GAE/mL different responses of different genotypes were evident: Palesio showed the lowest antioxidant capacity, while Gentil rosso was the most active genotype. Only at the highest dose 20 µg GAE/mL, the behavior of the different genotypes did not differ statistically (in fact all genotypes are labelled with the same letter “e”).

**Figure 4:**
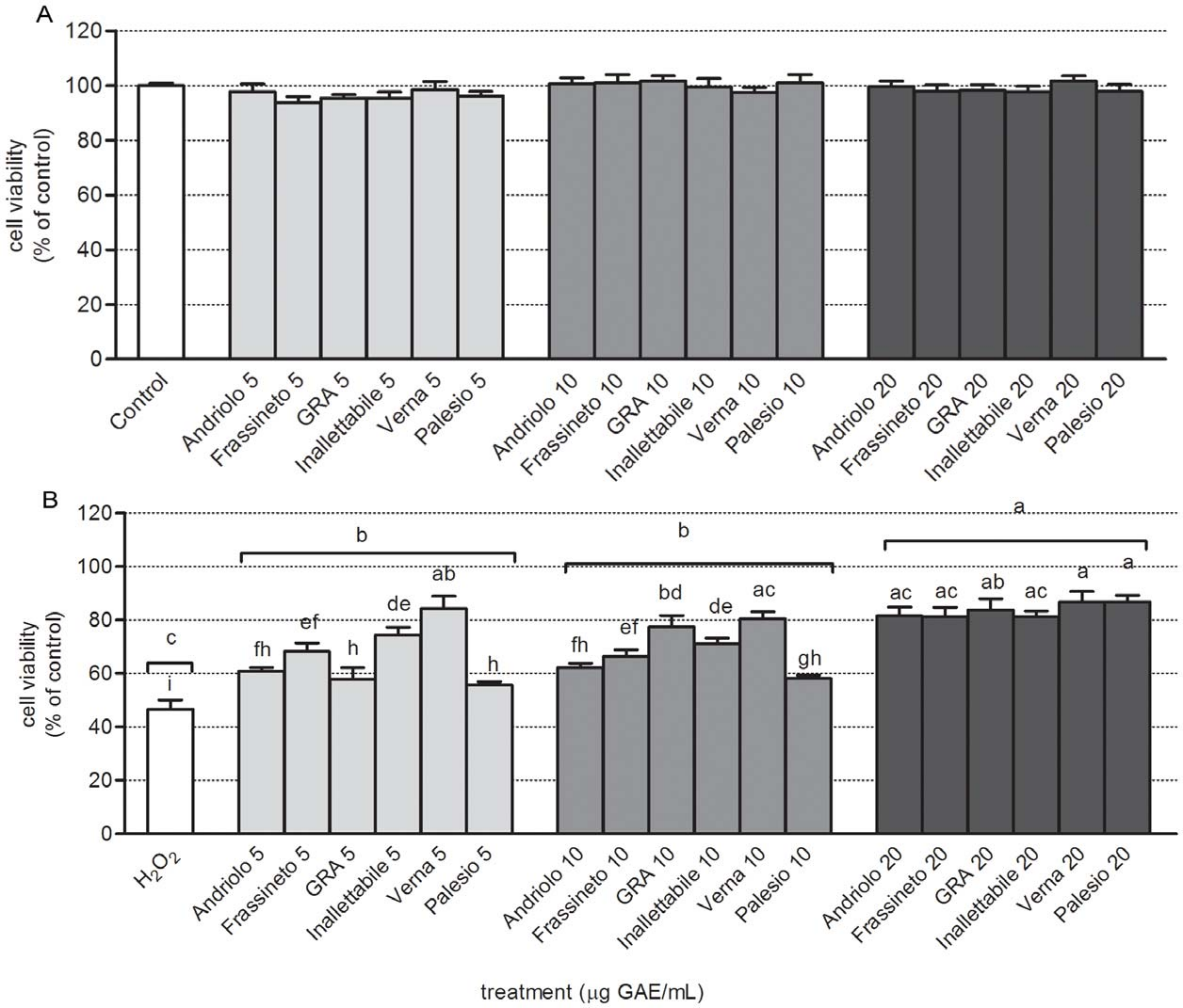
Evaluation of cell viability in primary cultures of neonatal rat cardiomyocytes. Cells were pre-treated with different concentrations (5, 10, 20 µg GAE/mL) of phenolic extracts of wheat varieties for 24 h after which oxidative damage was induced by treating cells with 100 µM H_2_O_2_ for 30 min. Cells were allowed to recover in fresh medium for additional 24 h after the stress and cell viability in the absence/presence of 100 µM H_2_O_2_ was assayed by MTT test as reported in Materials and Methods. Panel A represents cell viability levels in cells pre-treated with different phenolic extracts for 24 h, while panel B reports cell viability levels measured in cells pre-treated with different concentrations of phenolic exctracts for 24 h and stressed by H_2_O_2_ treatment. Data are reported as percent of control untreated cells and represent mean +/− SEM of 4 independent experiments, each in triplicate. Statistical analysis of differences was carried out by two way ANOVA followed by Fisher's LSD as post test. Different letters represent statistically significant differences (*p*<0.05) among wheat varieties at the same concentration (lower letters) and among different concentrations (upper letters).

The antiproliferative effect of wheat phenolic extract, evaluated by means of MTT test was dose-dependent and not affected by genotype (Fig. 3B). The effect of the dose was highly significant (*p*<0.001). The lowest dose was not significantly different from control, while the dose 10 µg GAE/mL represented the first significant effect observed with an average reduction of leukemic cell viability of 9.5% compared to control. The strongest effect was observed for the dose 20 µg GAE/mL, with a reduction of cell viability of 50.9% compared to control. This effect was significantly different compared to 10 µg GAE/mL. Overall, the reduction of viability of leukemic cells as a function of the dose was subjected to a logarithmic trend (R^2^ = 0.999). The genotype did not influence wheat extract antiproliferative effect (mean values are not statistically different) indicating that, on average, all genotypes exhibited the same type of action on leukemic cells viability. The interaction genotype x dose was not statistically significant, indicating that all genotypes maintain the same proportionality of the effect as a function of the dose: no effect at the dose 5 µg GAE/mL, an average reduction of 9.5% in cell viability at the dose 10 µg GAE/mL, and an average reduction of 50.9% at 20 µg GAE/mL. The decreased production of ROS is definitely connected to the observed effect on cell viability, indicating an antiproliferative action. This effect is considered to be specific against tumor cells, in fact untransformed cells, such as neonatal rat cardiomyocytes, treated with the same concentrations of extract, showed no significant changes in cell viability (Fig. 4A).

### Antioxidant activity and cytoprotective effects in primary cultures of neonatal rat cardiomyocytes

**Figure 5:**
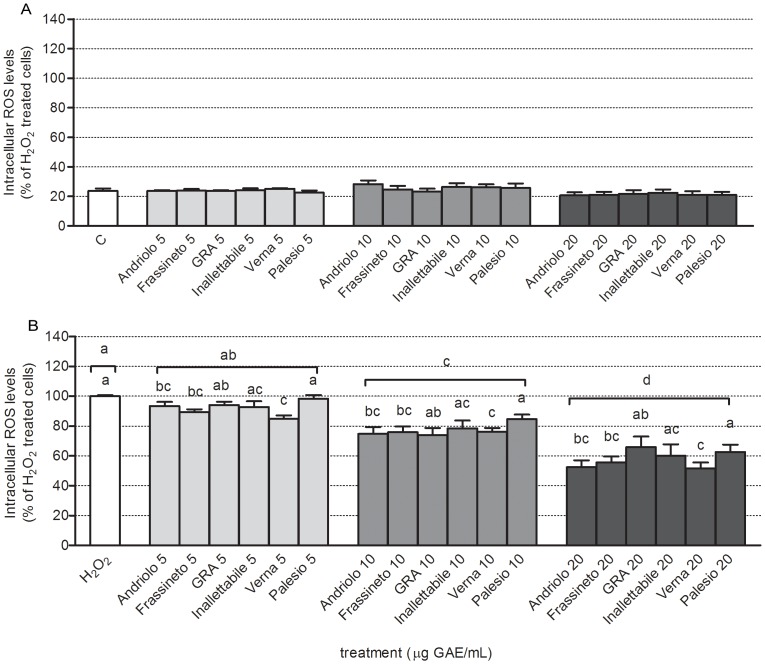
Evaluation of intracellular ROS production in primary cultures of neonatal rat cardiomyocytes. Cells were pre-treated with different concentrations of phenolic extracts of different wheat varieties (5, 10, 20 µg GAE/mL) for 24 h and intracellular ROS levels were measured in the absence/presence of 100 µM H_2_O_2_ as reported in Materials and Methods. Panel A represents basal intracellular ROS levels in cells pre-treated with different concentrations of phenolic extracts for 24 h, while panel B reports intracellular ROS levels measured in cells pre-treated with different concentrations of phenolic exctracts for 24 h and stressed by treatment with 100 µM H_2_O_2_ for 30 min. Data are reported as percent of H_2_O_2_-treated cells and represent mean +/− SEM of 4 independent experiments, each in triplicate. Statistical analysis of differences was carried out by two way ANOVA followed by Fisher's LSD as post test. Different letters represent statistically significant differences (*p*<0.05) among wheat varieties at the same concentration (lower letters) and among different concentrations (upper letters).

In order to mimic a state of oxidative stress, an aetiological factor of the most clinically relevant cardiovascular diseases, neonatal rat cardiomyocytes were treated with an oxidizing agent, 100 µM H_2_O_2_ for 30 min, and intracellular ROS levels were measured immediately after the stress was given. As presented in Figure 5B, the extracts of different wheat varieties analyzed exhibited a dose dependent (*p*<0.001) antioxidant activity in cardiac cells. The dose 5 µg GAE/mL partially differed from untreated stressed cells, with an average reduction in ROS levels of 7.9% compared to H_2_O_2_ treated cells. Raising the dose, a linear increase of the cellular effects (R^2^ = 0.986) was observed: at 10 µg GAE/mL treatment with different wheat extracts caused a 22.8% reduction of ROS levels, while at 20 µg GAE/mL the measured reduction was 41.9%, compared to H_2_O_2_ treated cells. Different genotypes exhibited, on average, different antioxidant activity: the old variety Verna resulted to be the most effective (labeled “a”), while the modern variety Palesio the less effective (labeled “c”). All other genotypes exerted intermediate antioxidant effect between that of Verna and Palesio.

The interaction genotype x dose was not statistically significant. This lack of significance indicates that all genotypes maintain the same proportionate effect depending on the dose.

For the evaluation of wheat extract cytoprotective effect, cell viability was measured 24 h after the induction of oxidative damage by means of H_2_O_2_. Results reported in Figure 4B showed that cell viability was significantly affected by the dose of wheat extract used for treatment (*p*<0.01). The lowest tested concentration (5 µg GAE/mL) caused an increase in cell viability of 20.9% which was significantly different from H_2_O_2_ treated cells, not pretreated with wheat extracts. Raising the dose caused an increase in cellular effects according to an exponential curve (R^2^ = 0.952): the dose 10 µg GAE/mL (increase of 22.4% compared to H_2_O_2_ treated cells) did not differ statistically from the dose 5 µg GAE/mL, while the dose 20 µg GAE/mL was found to differ from doses 5 and 10 µg GAE/mL, causing an increase of 36.9% in cell viability. All genotypes exhibited the same effects on cardiomyocytes viability. However, since the interaction genotype x dose was statistically significant, it is evident that at the different doses tested in our model system, individual extracts (genotypes) showed different cytoprotective effects (not only dose dependent).

### Principal component Analysis (PCA)

**Figure 6:**
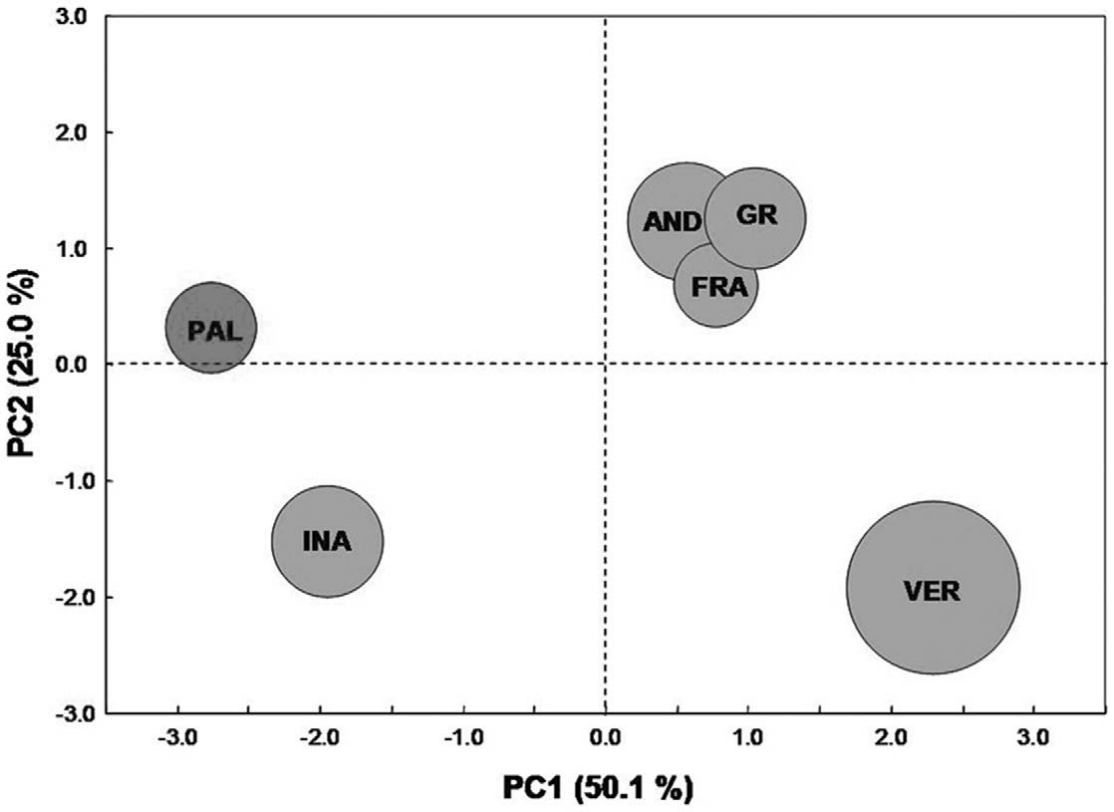
Principal component analysis based on seed anti-oxidant properties and seed extract activities at cellular level. The scatter plot reports the projection of cases (6 wheat genotypes) on the first two components PC1 and PC2 (accounting for 75.1% of total variability): the diameter of each balloon is proportional to the variability observed for the wheat accession.

The multiple intercorrelation between the different cellular assays and chemical charaterization of wheat extracts was determined by principal component analysis (PCA). PCA and factor analysis are the most widely used statistical methods to reduce the number of dimensions in data analysis and to investigate multiple intercorrelations between variables [28]. To define the number of principal components (PCs) explaining most of the total variance among a certain number of variables, different criteria can be used for choosing the number of dimensions that adequately describe the data matrix. One of them is the Kaiser criterion, based on the concept that a PC with an eigenvalue <1.0 has no legitimacy for the description of the total variance [31]. Out of the eleven extracted PCs, only the first and the second PC satisfied the Kaiser criterion, cumulatively explaining the 75.1% of the total variance (Table 5). The first PC accounted for 50.1% of the total variance and showed high loadings (>0.75) for FRAP activities of free and bound polyphenol fractions (FRAP_F, FRAP_B), DPPH activities of free and bound polyphenol fractions (DPPH_F, DPPH_B) and ROS level detection in cardiomyocytes and leukemia cell line (CR, LR). This result indicated a significant multiple correlation among the antioxidant activities measured both *in vitro* (DPPH, FRAP) and in cell lines (ROS detection) and with the first PC. Tentatively, the first PC was labeled as scavenging properties of wheat extracts. The second PC explained the 25.0% of the total variance and showed high positive loadings (>0.75) for the total number of bound polyphenol species and high negative loadings (<−0.75) for the ratio of total flavonoids on total polyphenols and MTT viability test on cardiomyocytes. Overall the positive branch of the second PC is tentatively labeled as abundance of polyphenol species (also the total number of free polyphenol species is partially loaded on the second PC positive branch), while the negative branch of the second PC indicated effects on cardiomyocyte viability which is associated with the ratio of total flavonoids on total polyphenols. A partial separation among cases (wheat varieties) in the scatterplot was achieved by combining the first and the second PC. As reported in Figure 6 the combination of PC1 and PC2 allowed clearly separate trajectories for Palesio, Verna and Inallettabile, while Gentil Rosso, Andriolo and Frassineto were closely aggregated in the North-East quadrant. Considering the placement of each wheat variety with respect to the first PC, the relative ranking for the overall scavenging activity was Verna > Gentil rosso > Frassineto > Andriolo > Inallettabile > Palesio. As regards the relative position of wheat varieties with respect to the second PC, Gentil rosso, Andriolo and Frassineto expressed the highest abundance of different polyphenol species, while the strongest positive effects on cardiomyocyte viability were ascribed to Inallettabile and Verna.

**Table 5:** Factor loadings of the eleven variables used in the principal component analysis on the first two principal components (PC1, PC2).

Variable	PC1	PC2
FRAP_F	0.795	−0.012
FRAP_B	0.781	−0.334
DPPH_F	0.791	−0.185
DPPH_B	0.778	0.118
F/P	0.616	−0.756
TNF	0.651	0.570
TNB	0.479	0.850
ML	−0.179	−0.347
RL	0.836	−0.036
MC	0.503	−0.785
RC	0.754	−0.250
Eigenvalue	3.506	1.747
% of variance	50.1	25.0

FRAP_F = FRAP activity of free polyphenol fraction; FRAP_B = FRAP activity of bound polyphenol fractions; DPPH_F = DPPH activities of free polyphenol fraction, DPPH_B = DPPH activities of bound polyphenol fraction; F/P = ratio of total flavonoids on total polyphenols, TNF = total number of free polyphenol species (including isomeric forms), TNB = total number of bound polyphenol species (including isomeric forms); ML = MTT viability test on leukemic cell line; RL = ROS test on leukemia cell line; MC = MTT viability test on cardiomyocytes; RC = ROS test on cardiomyocytes.

## Discussion

It is well established that the imbalance between the production of reactive oxygen species (ROS) and the neutralizing capacity of the antioxidant system generates a condition referred to as oxidative stress, a causative factor in the onset and development of several acute and chronic-degenerative diseases [32], [33], [34], [35], [36]. According to epidemiological data showing that diet plays a crucial role in the prevention of chronic diseases, such as cardiovascular diseases, cancer, diabetes, and neurodegeneration, there is an increasing interest in exploring and establishing new dietetic strategies aiming at the prevention/counteraction of oxidative damage and research is focused on the potential influence of exogenous antioxidants on cytoprotection [37], [38], [39].

The renewed interest in old common wheat varieties have caused the research interest to thoroughly analyse their nutraceutical components. Although other researchers have characterized the nutraceutical composition of some wheat kernels and have compared different old and modern cultivars [40], the results here reported represent a deeper insight into the correlation between antioxidant activity (FRAP and DPPH) and polyphenol/flavonoid content showing that antioxidant activity is mostly influenced by flavonoid (both bound and free) content and by the ratio flavonoids/polyphenols. Due to the very low amount of carotenoids, and the absence of tocopherols/tocotrienols and ascorbic acid in the tested ethanol extracts, the potential contribution of these bioactives to the antioxidant activity could be considered negligible.

This paper represents the first study on the analysis of the effect of different wheat varieties in cell culture systems. Of the plethora of effects attributed to plant flavonoids, both their potential to exert antiproliferative and cytotoxic activity towards aberrant cells and also their ability to act as antioxidants and cytoprotective agents has attracted attention as possible mechanisms contributing to the pathopreventive effects of fruit and vegetable-rich diets [41]. The health benefits derived from the consumption of cereals and cereal products, particularly wholegrains, are well established. Research has shown that wholegrain consumption help lower the risk of cardiovascular disease, ischemic stroke, type II diabetes and gastrointestinal cancer. In addition to dietary fiber, wholegrains contain many health promoting components such as vitamins, minerals, phytochemicals which include phenolic compounds. Phenolic compounds have antioxidant properties and can protect against degenerative diseases (*i.e*. heart diseases and cancer) in which ROS are involved. One example of diet based on wholegrains is represented by the Mediterranean diet, adherence to which has been associated to lower risk of chronic-degenerative diseases [42]. Most of the literature on plant phenolics focuses mainly on those present in fruit and vegetables, wines and teas. However, many phenolic compounds in fruit and vegetables (*i.e*. phenolic acids and flavonoids) are also reported in cereals [12], [43].

As evidenced by PCA, a multiple correlation between antioxidant capacity in cell free system (FRAP and DPPH), and ROS levels in both cardiac and leukemic cells was observed. All tested common wheat varieties maintained the antioxidant activity in biological systems when measured in term of ROS scavenging capacity. The effect exerted is dose-dependent according to a linear trend. Similar antioxidant effects were observed in different cell model systems, representative of transformed and non-transformed cells. In both cell model systems an effective antioxidant activity was detected at the lowest concentration tested (5 µg GAE/mL). The range of concentrations tested in this study is in accordance with those used in similar studies on polyphenol effects on cells parameters reported in literature [44], [45], [46]. Although extensively characterized in terms of nutraceuticals components only few reports are present in the literature on the effect of wheat polyphenolic extracts in biological systems. In a recent paper, Bonfili *et al*. [45] used extracts of wheat germ as a source of polyphenols, in order to assess the potential chemopreventive effect.

Within the experimental set of wheat varieties here analyzed a significant genotype effect on antioxidant activity was observed in both HL60 cell line and cardiomyocytes (Fig. 3A; Fig. 5B and Fig. 6). These effects could be ascribed to the peculiar phenolic pattern of each wheat variety. It is intriguing that clustering of different genotypes according to phenolic composition (Fig. 2) strongly overlaps the ordination of the same genotyope according to first principal components (PC), which represents the antioxidant activity (Fig. 6). On the whole, the most effective scavenging effect was observed for the old variety Verna, while Palesio and Inallettabile exibited the lowest one; the other old wheat varieties demonstrated intermediate antioxidant capacity (Fig. 6). Moreover, the potential correlation between the antioxidant activity of different extracts and the cytoprotective or antiproliferative effect was analysed. The decreased ROS intracellular levels is definitely connected to the effect on leukemia cell line viability, resulting in an antiproliferative action. In cardiomyocytes, the treatment with wholegrain extracts counteracted oxidative stress induced by H_2_O_2_ increasing cell viability at all concentration tested. As regards cell viability test, the effect of treatment with wheat phenolic extract is dose-dependent according to an exponential trend. The difference between the dose-response trend evidenced in ROS and MTT assay could be explained considering the intrinsic differences between the two tests: by means of a fluorescent probe, an instant picture of intracellular ROS levels is obtained, while the role of polyphenols in cell viability is a consequence of their ROS scavenging activity leading to protection of cardiomyocytes against H_2_O_2_ stress and to antiproliferative effect in cancer cells. Moreover, it is not surprising that ROS scavenging effects do not match with the corresponding effect on cell viability. Cell viability is modulated by several factors including ROS mediated signaling pathways requiring longer time to achieve the final effects (*i.e*. cell proliferation, cell death) as reported in several paper [47], [48], [49]. The lowest concentration tested (5 µg GAE/mL) resulted to be effective in cardiomyocyte protection/recovery after H_2_O_2_ treatment but was not able to decrease viability in HL60 cells. This could be explained taking into consideration the different levels/time of exposure of cells to oxidative stress: in cardiomyocytes polyphenols reacted with a single dose of oxidant, while in HL60 cells a continuous proliferative ROS production occurred, thus a higher dose of antioxidants could be required to affect intracellular ROS levels.

Considering the observed antioxidant and cytoprotective/antiproliferative properties of polyphenols, it is possible to hypothesize a protective action for these phytochemicals present in new and old wheat varieties and to define polyphenol containing cereals as “functional”. Although intervention studies in humans are required, *in vitro* studies are necessary and required for health claim substantiation regarding functional properties of the wheat varieties analyzed in this study.

The results reported in this paper show potential nutraceuticals effects of common wheats that can be transferred to a finished product, such as bread. In addition, the concentration tested in this study are usually found in wheat foodstuff as recently reported [50], [51]. Moreover, the increase of emerging diseases such as “gluten sensitivity” has been correlated to exposure to high level of gluten epitopes particularly abundant in modern varieties such as Glia-α9. The lower presence of Glia-α9 epitopes [52] along with richer quali-quantitative polyphenol contents [12], [53] suggest the potential use of old wheat varieties as raw material for developing wheat-derived foodstuffs with health promoting characteristics.

The presence of different nutraceutical components and their possible synergistic effect confirm the healthy role of cereals in human nutrition and, above all, underline the importance of a re-discovery of ancient varieties in the development of functional food aimed at the prevention of chronic-degenerative pathologies.
